# Aromatase gene and its effects on growth, reproductive and maternal ability traits in a multibreed sheep population from Brazil

**DOI:** 10.1590/S1415-47572009005000054

**Published:** 2009-09-01

**Authors:** Ana Maria Bezerra Oliveira Lôbo, Raimundo Nonato Braga Lôbo, Samuel Rezende Paiva

**Affiliations:** Departamento de Zootecnia, Universidade Federal do Ceará, Fortaleza, CEBrazil; 2 Embrapa Caprinos e Ovinos, Sobral, CearáBrazil; 3 Embrapa Recursos Genéticos e Biotecnologia, Brasília, DFBrazil

**Keywords:** body weight, lambing interval, PCR-RFLP, polymorphism, SNP, litter weight

## Abstract

We determined the polymorphism C242T of the aromatase gene (*Cyp19*) and its allelic frequency, as well as the effect of the variants on productive and reproductive traits in 71 purebred Santa Inês sheep, 13 purebred Brazilian Somali sheep, nine purebred Poll Dorset sheep, and 18 crossbred 1/2 Dorper sheep. The animals were genotyped using the PCR-RFLP technique. The influence of the animal's genotype on its performance or on the performance of its lambs was analyzed by the least square method. Another factor assessed was the importance of the animal's genotype in analysis models for quantitative breeding value estimates, and whether there were differences among the averages of breeding values of animals with different genotypes for this gene. In the sample studied, no AA individuals were observed; the AB and BB frequencies were 0.64 and 0.36, respectively. All Brazilian Somali sheep were of genotype BB. All 1/2 Dorper BB animals presented a lower age at first lambing, and the Santa Inês BB ewes presented a lower lambing interval. In these same genetic groups, AB ewes presented higher litter weight at weaning. This is evidence that BB ewes have a better reproductive performance phenotype, whereas AB ewes present a better maternal ability phenotype. However, in general, animals with genotype AB presented better average breeding values than those with genotype BB.

## Introduction

The aromatase cytochrome P450 enzyme is responsible for estrogen biosynthesis by conversion or aromatization of androgens into estrogens. Estrogen is a hormone with important endocrine, paracrine and autocrine activities, involved not only in the regulation of male and female reproduction, but also in other characteristics, such as fat deposition (Heine *et al.*, 2000; Jones *et al.*, 2000) and growth (Simpson *et al.*, 2000).

In the granulosa cells, aromatase is essential for folliculogenesis and, consequently, for oocyte quality. Aromatase is also related, through the aromatization of androgens into estrogens, with the estrus stimulus and the development of the mammary glands. In the ovarian follicles, the theca produces androgens that accumulate when the estrogen synthesis by the granulosa cells is inadequate. This accumulation seems to promote an inhibitory effect on the follicular structures, and consequently, the follicles become atretic and die. In many species, estrogen biosynthesis in the brain has been correlated to sexual behavior, such as mating responses and frequently a pronounced dimorphism between males and females (Simpson *et al.*, 1994).

Some studies performed in cattle have indicated that the estrogens play an important role in the maintenance of pregnancy (Wendorf *et al.*, 1983) and in the beginning of parturition (Hoffmann *et al.*, 1979; Thorburn and Challis, 1979). Estrogen synthesis begins with the mitochondrial cholesterol and androgen substrates, such as androstenedione, that are turned into estrogens by the enzyme aromatase (Conley and Hinshelwood, 2001).

The gene *Cyp19* that codifies the aromatase P450 enzyme in sheep has been mapped to bands q24-q31 of chromosome 7 (Payen *et al.*, 1995; Goldammer *et al.*, 1999). In exon 3, which is located in codon 69, a silent C/T transition was detected in several animals (Vanselow *et al.*, 1999). In sheep, *Cyp19* is transcribed from four different promoters (P1.1, P1.4, P1.5 and P2) that present organ-specific activities. P2 is mainly active in granulosa cells, P1.5 and P1.1 are active in the placenta, and P1.4 is active in the brain (Vanselow *et al.*, 1999; 2001).

Studies in humans have shown that a mutation in gene *Cyp19* seems to be associated with bone maturation and consequently with linear growth (Morishima *et al.*, 1995; Carani *et al.*, 1997). These studies established that estrogen, and not androgen, is responsible for the progress of bone maturation.

The aim of this study was to determine the presence and the allelic frequency of polymorphism C242T (AJ012153) in the aromatase gene in a multibreed sheep population from Brazil. In addition, the effects of the variants on growth, reproductive and maternal ability traits were assessed. The importance of the knowledge of the animal's genotype in the analysis models for estimating the quantitative breeding values of studied traits, and the existence of differences among the averages of these breeding values in animals with different genotypes for this gene were also evaluated.

## Materials and Methods

###  Blood samples

For DNA analysis, blood samples were collected from 71 purebred Santa Inês sheep (eight males and 63 females), 13 purebred Brazilian Somali sheep (all females), nine purebred Poll Dorset sheep (two males and seven females), and 18 crossbred 1/2 Dorper sheep (all females). These animals, 10 rams and 101 ewes, were raised in a herd (Gaasa Agropecuária Ltda.) located in Inhumas, Goiás, Brazil, under semi-intensive conditions. This herd is controlled by the Breeding Program for Meat Goats and Sheep (Programa de Melhoramento Genético de Caprinos e Ovinos de Corte - GENECOC), of Embrapa (Brazilian Agricultural Research Corporation) Caprinos e Ovinos.

###  Genotyping

DNA was extracted using the salting out protocol (Miller *et al.*, 1988). The PCR-RFLP technique was used to determine the presence and the allelic frequency of the C242T (AJ012153) polymorphism in the C*yp19* gene (aromatase). A 140 bp fragment of the gene was amplified using the primers described by Vanselow *et al.* (1999), in agreement with the sequence deposited in GenBank (AJ012153): primer 1-5' - CCA GCT ACT TTC TGG GAA TT - 3'; primer 2-5' - AAT AAG GGT TTC CTC TCC ACA - 3'. The polymorphism was determined by endonuclease digestion of the amplified fragment with the *Bsp143I* enzyme (isochizomer *DpnII*).

The PCR amplification reaction was carried out in an Eppendorf thermocycler, in a final reaction volume of 25 μL; each reaction contained 5 pmol of each primer, 1.5 U Taq DNA polymerase (Phoneutria), 100 μM of each dNTP, 2.5 mM MgCl_2_, 1X buffer, and 25, 50 or 100 ng ovine genomic DNA, depending on the sample. The amplification program consisted of an initial denaturation step (94 °C for 2 min), 35 denaturation (94 °C for 15 s), annealing (55 °C for 30 s) and elongation (70 °C for 2 min) cycles, followed by a final elongation step (70 °C for 5 min). The amplified products were submitted to electrophoresis on 4% agarose gels and visualized under an UV transilluminator, after staining with ethidium bromide.

A 10 μL aliquot of PCR products was digested in a total volume of 20 μL with 5 U of *Dpn*II (Biolabs) and 1X buffer for 14 h at 37 °C, followed by inactivation for 20 min at 65 °C. The digestion products were also electrophoresed on 4% agarose gel and visualized by ethidium bromide staining.

###  Statistical analyses

In order to determine the influence of the genotype of the animal on its performance or on the performance of its lambs (lambs of the 101 ewes), statistical analyses were made using the least square method of the GLM procedure of the SAS software (SAS Institute Inc., 1996). The genetic groups of the lambs tested were: Santa Inês (of Santa Inês ewes), 3/4 Santa Inês (of 1/2 Santa Inês ewes), 3/4 Dorper (of 1/2 Dorper ewes), and 7/8 Dorper (of 3/4 Dorper ewes). The growth traits analyzed were: birth weight (BW), weaning weight (WW), slaughter weight (SW), yearling weight (YW), weight gain from birth to weaning (GBW), weight gain from weaning to slaughter (GWS), and weight gain from weaning to yearling (GWY). These traits were evaluated in all animals (lambs and ewes). In ewes, the reproductive and maternal traits analyzed were: age at first lambing (AFL), lambing interval (LI), gestation length (GL), lambing date (LD; number of days between the beginning of the breeding season and the lambing), litter weight at birth (LWB), and litter weight at weaning (LWW).

Body weights and weight gains were adjusted for season and year of birth, sex (in the case of lamb performance), and birth type (single, twin, triple). BW, WW and GBW were adjusted for age classes (1 to 6) of the dam at lambing. WW, SW and YW were adjusted for age at measurement as covariate. These weight gains were also adjusted for the respective ages. AFL was adjusted for season and year of birth, animal birth type and lambing type (one male lamb, one female lamb, two male lambs, two female lambs, one male and one female lamb, three lambs regardless of sex). LI, LD, GL, LWB and LWW were adjusted for season and year of birth, lactation number and lambing type (as described above). LWW was also adjusted for the age at weaning as covariate.

Breeding values of the studied traits were estimated by the Derivative-Free Restricted Maximum Likelihood method (DFREML), using the MTDFREML software (Boldman *et al.*, 1995), with an animal model. The analyses were made including the animal genotype as fixed effect or not. These analyses were performed in two ways: either considering the trait measured in the genotyped animal, or considering it in the lambs of the genotyped ewe. The Likelihood Rate (LR) test (Rao, 1973) was used to determine whether models with genotype information were better than those without. LR was estimated as Lj/Li, where Lj was the restricted likelihood maximum for models without genotype information, and Li was the restricted likelihood maximum for models with genotype information. The -2 Log LR value was compared to the χ^2^ tabulated value, with one degree of freedom; the test was considered significant whenever the calculated value was higher than the tabulated value.

Finally, the differences among breeding values of the genotyped animals were determined by variance analysis.

## Results

**Figure 1:**
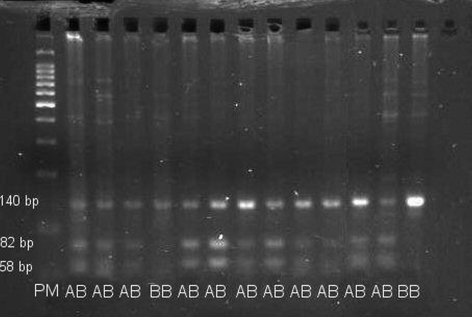
Analysis of RFLP polymorphism of the aromatase gene (*Cyp19*) in ovine. Non-digested PCR products are 140 bp in size (allele B). In the case of allele A, there are two fragments of 82 bp and 58 bp, respectively. PM = 100 bp Molecular Weight Ladder (Promega®). AB and BB are the deduced genotypes.

The PCR digestion products of 111 samples revealed only two genotypes for the *Cyp19* polymorphism: AB and BB. Genotype AB presents three fragments, of 140, 82 and 58 bp, respectively, whereas genotype BB presents only one fragment, of 140 bp. Figure 1 presents a sample of 13 genotyped animals.

The frequencies of genotypes AB and BB were 0.64 e 0.36, respectively. Table 1 shows the frequencies for alleles A and B, according to the genetic groups analyzed.

Table 2 presents the averages of the traits measured in the animals genotyped for gene *Cyp19*. Data on performance were available only for the animals of the 1/2 Dorper and Santa Inês genetic groups. No difference was observed regarding weight or weight gain among animals of different genotypes. However, some differences were observed for some traits in some genetic groups. In 1/2 Dorper, the BB animals presented lower AFL (p < 0.05). In the Santa Inês breed, animals with this same genotype also presented lower LI. For the maternal trait LWW, AB animals weaned heavier litters.

Table 3 presents the averages of traits measured in the lambs of genotyped ewes. Lambs of BB 1/2 Dorper ewes presented lower averages for BW, WW and GBW, while lambs of BB Santa Inês ewes presented lower averages only for WW.

Table 4 presents the values of the likelihood ratio test (LR) for models including or not the effect of the aromatase gene in the analysis for estimating the breeding values of the studied traits. For all traits and models considered, the utilization of the aromatase genotype effect was important. The inclusion of the genotype information allowed a better fitting of the analyses of the measured traits, both in the genotyped animal itself and in the lambs of genotyped ewes. A significant decrease (p < 0.01) in -2 Log L was found when the genotype information was included.

The comparison between the averages of breeding values estimated for growth, reproductive and maternal traits, according to the two genotypes observed for the aromatase gene, is presented in Table 5. Considering the breeding values estimated in genotyped animal, except for weaning weight (maternal effect), weight gain from weaning to slaughter, weight gain from weaning to yearling, age at first lambing and gestation length, there were differences in the averages of breeding values.

Regarding growth traits, the animals with genotype AB presented, on average, higher breeding values than those with genotype BB. Similarly, the lambs of ewes with genotype AB presented higher breeding values than those of mothers with genotype BB, except for GWS.

With regard to reproductive traits, there were differences only in LI and LD. The animals with genotype AB presented better breeding values for LI (lower) and worse breeding values for LD (higher). Ewes with genotype AB presented a higher maternal ability than those with genotype BB. Their values for LWB and LWW were superior by 0.099 kg and 0.319 kg, respectively. This trend was confirmed in the analysis that considered the effect of the maternal genotype on the breeding values of their lambs. The expected progeny differences regarding BW, WW and GBW were, respectively, 0.06 kg, 0.08 kg and 1.79 g/day higher for lambs of AB ewes than for lambs of BB ewes.

## Discussion

Coincidently, all rams of the herd sampled in this work had genotype AB. There were no AA animals in the analyzed sample, probably due to the low frequency of allele A. Vanselow *et al.* (1999) studied purebred ovine animals from Europe and identified three genotypes, with a frequency of 0.74 for allele A and 0.26 for allele B in Hungarian Merino animals, and a frequency of 1.00 for allele A (none for allele B) in Lacaune animals. To our best knowledge, besides the study of Vanselow *et al.* (1999), there is no other report in the literature on aromatase gene frequencies in ovine. In all animals sampled, except Brazilian Somali, polymorphism for the aromatase gene was observed.

High aromatase activity in trophoblast of preimplantation pig embryos is associated with maternal recognition of pregnancy and the occurrence of superficial implantation in pigs, sheep, cows, roe deer, ferrets, cats, rabbits and a plains viscacha (Gadsby *et al.*, 1980).

In male mice, Wang *et al.* (2001) observed that elevated *Cyp19* expression was followed by an increase in the intratesticular levels of estradiol. These authors concluded that aromatase seems to be a physiological target of Dax-1 (an orphan nuclear receptor that represses transcription by steroidogenic factor-1, SF-1) in Leydig cells, and increased *Cyp19* expression may account, in part, for infertility and Leydig cell hyperplasia (LCH) in *Dax1*-deficient mice.

Gestagens and estrogens are important regulators of pregnancy and parturition. Vanselow *et al.* (2004) reported that in cattle and sheep *Cyp19* transcripts were found at high concentrations in the placenta and at a very low concentration in the corpus luteum. According to Luo and Wiltbank (2006), in bovine, *Cyp19A1* (aromatase) is regulated by multiple pathways, including estrogen receptors and cAMP/protein kinase A induced by FSH hormone activation in granulosa cells. These inter and intracellular regulatory mechanisms may be critical for normal follicle growth and dominant follicle selection.

All these pieces of evidence allow concluding that the polymorphism produces differences in aromatase activity, justifying the differences observed in reproductive performance (AFL and LI) and in maternal ability (LWW) of some genetic groups studied here. Aromatase inhibition rapidly decreases both appetitive and consummatory aspects of male sexual behavior (Cornil *et al.*, 2006; Roselli *et al.*, 2006). Therefore, such enzyme activity differences among animals could produce a difference in AFL and in LI. On the other hand, it is known that traits measured until weaning are subject to strong maternal influence. Thus, our analyses of weight and weight gains until weaning corroborate the findings for LWW, *i.e.*, that AB aromatase gene ewes tend to present greater maternal ability. However, in Black-and-White and Jersey cattle, Jedrzejczak *et al.* (2006) did not observe any association between the *CYP19*-*Pvu*II polymorphism and milk production traits.

The better fitting of models with the inclusion of the aromatase gene genotype suggests that whenever information about the animal genotype is available should be included in the models for estimating genetic parameters and breeding values. It is important to point out that the small number of genotyped animals in this study did not permit greater considerations about the estimated parameters. However, the inclusion of the genotype was important for the comparative analysis. Thus, as a larger number of genotyped animals becomese available from future studies, this information can and should be included in the analysis models for estimates of quantitative breeding values.

Overall, differences among breeding values were observed according to the genotype for the aromatase gene. Using this information could improve the breeding values estimates. The use of the animal's genotype or of the genotype of its mother in the analysis will depend on the amount of available information or of the trait itself. We suggest that traits under larger maternal influence should be analyzed using the information of the mother's genotype. On the other hand, traits that are more subject to the effect of the individual's genes should be evaluated by including the information of this individual's genotype. It is also worth pointing out that the use of the mother's genotype multiplies the data available for analysis, once a ewe has several lambs, and this information can be used in the analyses of the traits of these lambs.

In this study, the genotype was included as fixed effect, so in future investigations other analyses could be made, as suggested by Muir (2007), combining mixed models utilizing a second random genetic effect for genotype, once there is sufficient recorded information on genotype and phenotype.

The heterozygotes for the aromatase gene showed, on average, better breeding values than the BB homozygotes. The estimated breeding values are due to additive genetic action. However, as in this population there is a participation of many breeds and their crossbreeding, it is possible that non-additive genetic effects were present in these estimates. The combination of breeds promotes higher heterosis, due to the increase of heterozygosis in the population. This could explain the superiority of heterozygote animals.

The results of this study point to a so far not elucidated difference among sheep with different genotypes for the aromatase gene. Future investigations may reveal whether this polymorphism is responsible for differences in the performance of animals and thus help in the selection of superior animals.

## Figures and Tables

**Table 1:** Allelic frequencies of the breeds studied.

Genetic group	Number of animals	Frequency of allele A	Frequency of allele B
1/2 Dorper	18	0.194	0.806
Poll Dorset	9	0.390	0.610
Santa Inês	71	0.400	0.600
Brazilian Somali	13	0.000	1.000

**Table 2:** Least square means for traits measured in animals with genotypes AB and BB for the aromatase gene.

		Genotype AB			Genotype BB	
Trait	Genetic group	N	Mean ± standard error		N	Mean ± standard error
BW (kg)	1/2 Dorper	7	3.43 ± 0.29		11	2.86 ± 0.32
	Santa Inês	44	3.69 ± 0.45		12	3.21 ± 0.51
WW (kg)	1/2 Dorper	7	12.75 ± 2.11		11	11.77 ± 2.53
	Santa Inês	43	17.01 ± 1.27		11	16.38 ± 1.41
YW (kg)	Santa Inês	16	36.25 ± 2.51		5	42.00 ± 2.73
GBW (kg)	1/2 Dorper	7	0.190 ± 0.038		11	0.184 ± 0.046
	Santa Inês	42	0.231 ± 0.021		11	0.224 ± 0.024
GWY (kg)	Santa Inês	15	0.069 ± 0.008		4	0.084 ± 0.009
AFL (day)	1/2 Dorper	5	549.42 ± 26.12^a^		8	361.84 ± 37.52^b^
	Santa Inês	18	526.44 ± 23.64		9	556.17 ± 23.46
LI (day)	1/2 Dorper	6	231.95 ± 13.60		9	221.73 ± 9.42
	Santa Inês	58	246.48 ± 15.07^a^		9	218.75 ± 19.01^b^
LD (day)	Santa Inês	18	162.43 ± 6.13		3	178.89 ± 10.52
GL (day)	1/2 Dorper	11	149.30 ± 0.96		17	148.03 ± 1.02
	Santa Inês	77	150.87 ± 0.88		19	149.82 ± 1.19
LWB (kg)	1/2 Dorper	11	5.44 ± 0.32		17	5.38 ± 0.34
	Santa Inês	77	5.96 ± 0.20		19	5.94 ± 0.27
LWW (kg)	1/2 Dorper	6	24.96 ± 1.31^a^		14	19.88 ± 1.40^b^
	Santa Inês	56	20.15 ± 0.93^a^		13	17.89 ± 1.32^b^

^a,b^Means with different superscripts are statistically different (p < 0.05; *t* test); BW = birth weight, WW = weaning weight, YW = yearling weight, GBW = weight gain from birth to weaning, GWY = weight gain from weaning to yearling, AFL = age at first lambing, LI = lambing interval, GL = gestation length, LD = lambing date, LWB = litter weight at birth, LWW = litter weight at weaning; N = number of observations.

**Table 3:** Least square means for traits measured in lambs of ewes genotyped for the aromatase gene.

		Genotype AB			Genotype BB	
Trait	Genetic group	N	Mean ± standard error		N	Mean ± standard error
BW (kg)	1/2 Dorper	17	4.25 ± 0.21^a^		38	3.22 ± 0.13^b^
	1/2 Santa Inês	1	3.82 ± 0.69		28	3.36 ± 0.17
	3/4 Dorper	17	3.41 ± 0.26		14	3.34 ± 0.27
	Santa Inês	126	3.89 ± 0.10		22	3.61 ± 0.17
WW (kg)	1/2 Dorper	12	16.93 ± 0.69^a^		30	14.42 ± 0.47^b^
	1/2 Santa Inês	1	14.59 ± 5.02		22	13.04 ± 1.37
	3/4 Dorper	8	16.51 ± 1.51		11	13.09 ± 1.62
	Santa Inês	91	15.54 ± 0.41^a^		16	13.86 ± 0.81^b^
SW (kg)	Santa Inês	11	30.54 ± 1.17		2	32.74 ± 2.39
YW (kg)	Santa Inês	12	36.54 ± 4.01		4	37.91 ± 8.14
GBW (kg)	1/2 Dorper	12	0.264 ± 0.012^a^		30	0.224 ± 0.008^b^
	1/2 Santa Inês	1	0.191 ± 0.092		22	0.190 ± 0.021
	3/4 Dorper	8	0.274 ± 0.033		11	0.198 ± 0.035
	Santa Inês	91	0.236 ± 0.009		16	0.211 ± 0.017
GWS (kg)	Santa Inês	11	0.202 ± 0.010		2	0.240 ± 0.020
GWY (kg)	Santa Inês	10	0.056 ± 0.009		4	0.053 ± 0.016

^a,b^Means with different superscripts are statistically different (p < 0.05; *t* test); BW = birth weight, WW = weaning weight, SW = slaughter weight, YW = yearling weight, GBW = weight gain from birth to weaning, GWS = weight gain from weaning to slaughter, GWY = weight gain from weaning to yearling; N = number of observations.

**Table 4:** Logarithm values of the likelihood function (-2 Log L) and values for likelihood rate (LR) test for the studied traits, according to the respective model.

Model/trait measured in genotyped animal	Without genotype effect -2 Log L	With genotype effect -2 Log L	-2 Log LR
BW, WW and YW	208.8689	175.4608	33.41**
GBW and GWY	389.1728	327.0870	62.08**
AFL, LI and GL	446.9582	410.3368	36.62**
LD	62.7478	54.9946	7.75**
LWB and LWW	148.2612	128.5329	19.72**

Model/trait measured in lambs of genotyped ewes	Without maternal genotype effect -2 Log L	With maternal genotype effect -2 Log L	-2 Log LR

BW, WW and SW	543.1798	510.7648	32.41**
BW, WW and YW	540.6258	506.3233	34.30**
GBW and GWS	1247.8714	1173.4227	74.45**
GBW and GWY	1209.9369	1142.5747	67.36**

**p < 0.01; BW = birth weight; WW = weaning weight; SW = slaughter weight; YW = yearling weight; GBW = weight gain from birth to weaning; GWS = weight gain from weaning to slaughter; GWY = weight gain from weaning to yearling; AFL = age at first lambing; LI = lambing interval; GL = gestation length; LD = lambing date; LWB = litter weight at birth; LWW = litter weight at weaning.

**Table 5:** Comparison between averages of breeding values estimated for growth, reproductive and maternal traits of a multibreed population of sheep, according to the genotype for the aromatase gene.

	Genotypes	
Trait	AB	BB
Breeding values estimated in genotyped animal		
Birth weight - direct effect (kg)	0.205^a^	0.003^b^
Birth weight - maternal effect (kg)	0.015^a^	-0.085^b^
Weaning weight - direct effect (kg)	0.722^a^	-0.048^b^
Weaning weight - maternal effect (kg)	-0.072^a^	-0.237^a^
Yearling weight (kg)	1.060^a^	-0.754^b^
Slaughter weight (kg)	1.000^a^	-0.778^b^
Weight gain from birth to weaning - direct effect (g/day)	8.182^a^	2.907^b^
Weight gain from birth to weaning - maternal effect (g/day)	1.887^a^	-4.043^b^
Weight gain from weaning to slaughter (g/day)	-3.335^a^	-2.505^a^
Weight gain from weaning to yearling (g/day)	1.685^a^	0.422^a^
Age at first lambing (day)	1.605^a^	1.375^a^
Lambing interval (day)	-5.653^a^	-3.673^b^
Lambing date (day)	0.2790^a^	0.0646^b^
Gestation length (day)	-0.0825^a^	-0.0934^a^
Litter weight at birth (kg)	0.128^a^	0.029^b^
Litter weight at weaning (kg)	0.282^a^	-0.037^b^

Breeding values estimated in lambs of genotyped ewes		

Birth weight - direct effect (kg)	0.190^a^	-0.030^b^
Birth weight - maternal effect (kg)	0.034^a^	-0.082^b^
Weaning weight - direct effect (kg)	0.745^a^	-0.027^b^
Weaning weight - maternal effect (kg)	-0.025^a^	-0.185^b^
Yearling weight (kg)	1.181^a^	-0.704^b^
Slaughter weight (kg)	1.043^a^	-0.539^b^
Weight gain from birth to weaning - direct effect (g/day)	9.670^a^	4.722^b^
Weight gain from birth to weaning - maternal effect (g/day)	1.750^a^	-1.828^b^
Weight gain from weaning to slaughter (g/day)	-2.986^b^	0.619^a^
Weight gain from weaning to yearling (g/day)	2.129^a^	0.687^b^

Same letters in same row indicate that there is no statistical difference (p > 0.05; *t* test).
